# Spot quantification in two dimensional gel electrophoresis image analysis: comparison of different approaches and presentation of a novel compound fitting algorithm

**DOI:** 10.1186/1471-2105-15-181

**Published:** 2014-06-11

**Authors:** Jan M Brauner, Teja W Groemer, Armin Stroebel, Simon Grosse-Holz, Timo Oberstein, Jens Wiltfang, Johannes Kornhuber, Juan Manuel Maler

**Affiliations:** 1 Department of Psychiatry and Psychotherapy, Friedrich-Alexander-University of Erlangen-Nuremberg, Schwabachanlage 6, 091054 Erlangen, Germany; 2 Department of Psychiatry and Psychotherapy, University of Duisburg-Essen, Virchowstrasse 174, 45147 Essen, Germany; 3 CERES GMBH, Evaluation & research, Marie-Curie-Strasse 8, 79539 Lörrach, Germany

**Keywords:** Two dimensional gel electrophoresis, Beta amyloid, Image analysis, Spot quantification

## Abstract

**Background:**

Various computer-based methods exist for the detection and quantification of protein spots in two dimensional gel electrophoresis images. Area-based methods are commonly used for spot quantification: an area is assigned to each spot and the sum of the pixel intensities in that area, the so-called volume, is used a measure for spot signal. Other methods use the optical density, i.e. the intensity of the most intense pixel of a spot, or calculate the volume from the parameters of a fitted function.

**Results:**

In this study we compare the performance of different spot quantification methods using synthetic and real data. We propose a ready-to-use algorithm for spot detection and quantification that uses fitting of two dimensional Gaussian function curves for the extraction of data from two dimensional gel electrophoresis (2-DE) images. The algorithm implements fitting using logical compounds and is computationally efficient. The applicability of the compound fitting algorithm was evaluated for various simulated data and compared with other quantification approaches. We provide evidence that even if an incorrect bell-shaped function is used, the fitting method is superior to other approaches, especially when spots overlap. Finally, we validated the method with experimental data of urea-based 2-DE of Aβ peptides andre-analyzed published data sets. Our methods showed higher precision and accuracy than other approaches when applied to exposure time series and standard gels.

**Conclusion:**

Compound fitting as a quantification method for 2-DE spots shows several advantages over other approaches and could be combined with various spot detection methods.

The algorithm was scripted in MATLAB (Mathworks) and is available as a supplemental file.

## Background

2-dimensional gel electrophoresis (2-DE) is a powerful tool in proteomics and software-based analysis of gel images is an important step in the process. Various computer-based methods have been proposed for the detection of protein spots in 2-DE images [1]. Recent developments involve segmentation-oriented methods, which are based on the watershed transformation [2,3] or surface-oriented methods [4]. However, a general concern regarding current spot detection and quantification algorithms is that no method is fully automated, because they require the use of user-defined parameters [1]. Often, more complex methods require additional input parameters [5] and thus can be difficult to implement for a user less experienced in image processing. This difficulty and other factors might be the reasons why the automated image analysis of electrophoretic data is not yet standard in all fields of research. Despite numerous solutions for automated 2-DE analysis have been introduced [1], the majority of these approaches focus on spot detection rather than on spot quantification. The amount of marked protein in an electro chemiluminescent spot is proportional to the number of photons detected by the camera in the respective pixels, which is proportional to the intensities of those pixels. Therefore, to obtain measures for protein quantities, one has to determine the overall signal amount of every spot. Common methods for spot quantification are area-based approaches that quantitate spots based on the areas they occupy in the image. There are various published methods for determining these spot areas [3,4,6,7], many of which make use of the watershed transformation. Spots can be quantitated by various area-related measures and many methods suggest the use of volume, i.e. the sum of pixel intensities within the respective areas. However, as we show in this paper, area-based approaches face certain limitations, especially in analyzing superimposed spots. The optical density (OD), i.e. the intensity of the most intense pixel of a spot, can also be used to quantify spots [8]. A different quantification approach is to find a fitted function for each spot and calculate the volume under the surface (VUS) of the function curve. Fitting of 2D-Gaussian function curves has been used for the quantification of 2-DE spots for a long time [9,10], despite the fact that the adequate functions to model 2-DE spots continue to be contentious [1,11]. Recent developments include a method to improve the applicability of Gaussian functions for saturated spots [12] or the use of 2D-gaussian function curves in the creation of synthetic gel images for the evaluation of image analysis algorithms [13]. In this study, we propose a simple algorithm that fits Gaussian functions to detected spots. We introduce the novel concept of compound fitting, i.e. the simultaneous fitting of neighboring groups of spots, for computationally efficient resolution of overlapping spots. Furthermore, we conduct the, to our knowledge, first structured comparison between different spot quantification approaches, namely the use of optical density of a spot, area-based quantification and Gaussian function fitting. The proposed compound fitting method is a highly accurate system for spot quantification and we demonstrate its superior performance relative to other methods for both synthetic and real data.

Regarding real 2-DE data, we chose beta amyloid (Aβ) peptides as our primary study system for two reasons: Firstly, 2-DE is a valuable tool in the analysis of Aβpeptides. Although it is difficult to separate low concentrated beta amyloid (Aβ) peptides based only on their almost identical masses, their high content of hydrophobic amino acids has been used to efficiently separate Aβ species with single amino acid differences in urea gels according to their hydrophobicity [14]. However, efficient separation can only be achieved using 2-dimensional gel electrophoresis because many species have identical hydrophobicities but different isoelectric points [15].The use of 2-dimensional electrophoretic methods has the advantage of displaying more than 30 different peptides at very low concentrations [15], that in surface enhanced laser desorption/ionization time-of flight mass spectroscopy are not detectable [16]. Secondly, exact quantification is especially important in the field of Aβ peptides, because it is necessary not only for the analysis of differential expression in different experimental conditions, but also for the use of Aβpeptides as biomarkers. The quantification of single Aβ peptide species and their ratios, e.g. Aβ_1-42_ and Aβ_1-40_ in particular, is valuable for the neurochemical diagnosis of Alzheimer’s dementia [16]. High-quality quantification is crucial to use these peptides as biomarkers [17].

## Methods

### Spot detection and compound fitting algorithm

**Figure 1:**
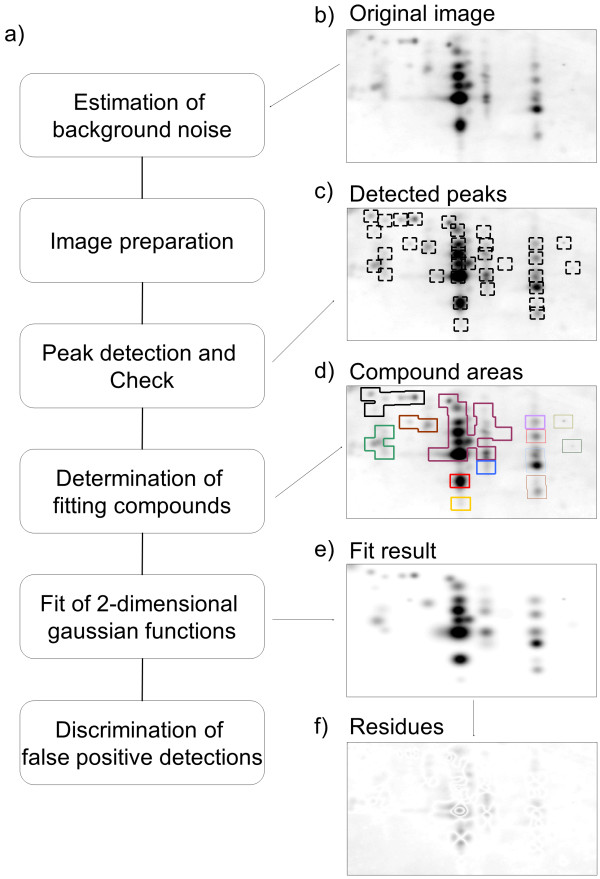
**Schematic illustration of the image analysis procedure. (a)** Flowchart of the algorithm. **(b)** Representative Image of 2-dimensional urea gel electrophoresis of Aβ peptides from human plasma. **(c)** Detected spots outlined. **(d)** Fitting compounds are indicated. **(e)** Fit result (image calculated from the fitted functions). **(f)** Residual image; The same level of contrast is used for all images **(b)** - **(f)**.

Our algorithm comprises two major components: spot detection/identification and spot quantification. The flowchart of the algorithm is shown in Figure 1a. Spot detection (step 2 and 3) was performed using a simplified version of an established method [18,19]. The algorithm involves a user-defined parameter *w*, which is used as the minimum allowed distance between peaks. A peak is defined as the most intense pixel of a spot. For 2-DE spots, the peak is usually in the center of a spot. Thus, *w* must be chosen to be smaller than the distance between two spots but sufficiently large to prevent the detection of several peaks on one spot. For the application of the algorithm on real data, the following user-friendly and intuitive method for selecting the value of *w* may be employed: the user is asked to click on the peaks of the two clearly identifiable spots with the smallest inter-peak distance (IPD).

Based on this input, *w* is calculated as

$$w=\left[\frac{\mathrm{IPD}}{2}\right],$$

where [ ] is the Gaussian floor function because *w* must be an integer. If *w is* set/calculated too small, several peaks might be detected on top of a broad spot. If *w* is estimated too large, the detection might fail to resolve overlapping spots.

#### Estimation of background noise

The first step of the spot detection algorithm is the estimation of the standard deviation (SD) of the image noise *σ*_*ns*_, which is performed in an area where only background signal is detected. Usually such an area without protein spots can be found in any typical 2-DE image, often near the edges. We used the following approach to implement the estimation.

The entire image A(x,y) is rastered with a quadratic *w* by *w* pixel grid. For every pixel (x,y), the SD *σ*_*area*_(*x*, *y*) and the mean value *μ*_*area*_(*x*, *y*) of the surrounding *w* by *w* area’s pixel intensities are calculated. *μ*_*area*_(*x*, *y*) of a pixel in a background area should be less than the mean of all *μ*_*area*_(*x*, *y*), because the majority of a gel image is occupied with background. Thus, the mean value of *σ*_*area*_(*x*, *y*) that also satisfies

$$\mu_{\mathrm{area}}\left(x,y\right)<\mathrm{mea}n_{i,j}\left(\mu_{\mathrm{area}}\left(i,j\right)\right)\;\mathrm{is}\;\mathrm{taken}\;\mathrm{as}\;\sigma_{\mathrm{ns}}.$$

#### Image preparation

Like any digital image, 2-DE images suffer from various types of noise and imperfections. We implemented noise reduction and image restoration for the spot detection procedure, whereas the function fit was performed using the original image A(x,y).

The following operations were performed:

– the local background was modeled using a boxcar average over a square region of edge length 2 · *w* + 1,

– random digital noise was suppressed by convoluting the image using a Gaussian surface of standard deviation $\sqrt{2}$ and

– the restored image was calculated by subtracting the background image from the noise-reduced image.

These steps can be performed in one convolution. The restored image, which is the basis for further spot detection, was calculated as

$$A_{\mathrm{res}}\left(x,y\right)=\sum_{i=-w}^{w}\sum_{j=-w}^{w}A\left(x-i,y-j\right)K\left(i,j\right),$$

where the convolution kernel K, normalization constant *K*_0_ and factor B are given as follows:

$$K\left(i,j\right)=\frac{1}{K_{0}}\left[\frac{1}{B}\exp\left(-\frac{i^{2}+j^{2}}{4}\right)-\frac{1}{{\left(2w+1\right)}^{2}}\right]$$

$$K_{0}=\frac{1}{B}{\left[\sum_{i=-w}^{w}\exp\left(-\frac{i^{2}}{2}\right)\right]}^{2}-\frac{B}{{\left(2w+1\right)}^{2}}$$

$$B={\left[\sum_{i=-w}^{w}\exp\left(-\frac{i^{2}}{4}\right)\right]}^{2}$$

#### Peak detection

In the following section, we will use the term 'peak’ to refer to the most intense pixel of a protein spot, usually in the center of the spot. In the restored image, a particular pixel was considered to be the location of a peak if it met two conditions:

a) It was the brightest pixel within the distance of *w*.

b) Its intensity was greater than a threshold defined by *σ*_*ns*_ and the local factor *μ*_*w*_, which is calculated as the mean intensity of all pixels within the distance of *w*.

a) This criterion is efficiently computed with a grayscale dilation of the image with a flat structuring element of circular shape and radius *w*.

The dilated image *A*_*dil*_ is given as

Consequently, pixels (*x*_*p*_, *y*_*p*_) that satisfy

b) After the peak candidates have been identified, a pixel (*x*_*p*_, *y*_*p*_) is considered a peak if it satisfies

$$A_{\mathrm{dil}}\left(x,y\right)=\max_{\left(i^{2}+j^{2}\leq w^{2}\right)}\left(A_{\mathrm{res}}\left(x+i,y+j\right)\right).$$

$$A_{\mathrm{res}}\left(x_{p},y_{p}\right)=A_{\mathrm{dil}}\left(x_{p},y_{p}\right)$$

are potential peak candidates.

$$A\left(x_{p},y_{p}\right)>\mu_{w}\left(x_{p},y_{p}\right)+t\cdot \sigma_{\mathrm{ns}},$$

where

$$\mu_{w}\left(x,y\right)=(\sum_{i^{2}+j^{2}\leq w^{2}}A\left(x+i,y+j\right))/\left(\underset{i^{2}+j^{2}\leq w^{2}}{\sum }1\right).$$

Based on our experience, t = 10 yields good results for real 2-DE images. For t < 10, a higher rate of false-positive detections is expected, especially for relatively large image sizes. If t is set too large, the rate of false negatives might increase.

#### Determination of the fitting compounds

For every spot, a Gaussian function is fitted in 4 parameters (for the equation, see next paragraph). For an assumed number of 50 spots per image, fitting the entire image at once would mean calculating the (nonlinear) influence of 200 parameters for every pixel, which is not possible within a reasonable computation time. To solve this problem, we used a compound fitting process that only fits neighboring groups of spots within the surrounding area of pixels simultaneously.

The general concept is that the spots of an image are divided into compounds - in such a way that the spots from one compound do not affect the pixels of another compound area. The defined groups of spots can then be fit individually within their respective compound areas, thereby significantly reducing the necessary computation time.

In the following, the term “compound” will be used for a group of spots that are fitted at the same time. The term “compound area” refers to the area that surrounds all the spots from one compound, comprising all the pixels that are significantly influenced by the spots and therefore the area that must be considered to be able to perform a proper fit.

The compound area around a single spot is a quadrat of edge length *d*. The minimum size of *d* is calculated in the results section. If two spots are close to each other in such a way that their compound areas would overlap, the overlapping area is influenced by both spots, and therefore, these spots must be fit at the same time. In this case, their individual compounds need to be fused, which means that if any two peaks P and Q satisfy |*x*_*P*_ - *x*_*Q*_| ≤ *d* and |*y*_*P*_ - *y*_*Q*_| ≤ *d*, they always belong to the same compound. This definition is compatible with the fact that in a single fitting compound, pairs of peaks can occur that do not satisfy this condition. For example, if there are three peaks P, Q, and R with

$$\left|x_{P}-x_{Q}\right|\leq d,\;\left|y_{P}-y_{Q}\right|\leq d,\;\left|x_{Q}-x_{R}\right|\leq d\;\mathrm{and}\;\left|y_{Q}-y_{R}\right|\leq d,$$

there will be only one fitting compound for all three spots, even if |*x*_*P*_ - *x*_*R*_| > *d*.

In this case, peak P and Q are called connected, whereas P is called connected to R via Q.

A reasonable approach for the determination of the compounds is to calculate the adjacency matrix:

The peaks are numbered, where N is the number of peaks/spot.

The matrix *I*_1,*norm*_ has a size of *N* × *N* and is defined as

$$I_{1,\mathrm{norm}}\left(i,j\right)=\{\begin{array}{l} 1,\;\mathrm{if}\;\left|x_{i}-x_{j}\right|\leq d\;\mathrm{and}\;\left|y_{i}-y_{j}\right|\leq d\\ 0,\;\mathrm{otherwise} \end{array}\;\},$$

where *x*_*i*_, *y*_*i*_, *x*_*j*_, *y*_*j*_ are the peak coordinates of the respective spots.

The subscripts indicate that *I*_1,*norm*_ is the adjacency matrix of first order and it is normalized so that the entries are either 0 or 1. The entry of *I*_1,*norm*_ at a certain position (P,Q) has the value 1, if the peaks P and Q are connected. Otherwise, the entry has the value 0. Of course, this constraint also means that all diagonal elements are 1.

Next the square of *I*_1,*norm*_ is calculated

$$I_{2}={I_{1,\mathrm{norm}}}^{2}$$

and thus

$$I_{2}\left(i,j\right)=\sum_{k=1}^{N}I_{1,\mathrm{norm}}\left(i,k\right)\cdot I_{1,\mathrm{norm}}\left(k,j\right).$$

*I*_2,*norm*_ is a matrix identical to *I*_2_ but with all elements larger than 1 set to 1. Thus,

$$I_{2,\mathrm{norm}}\left(i,j\right)=\{\begin{array}{l} 1,\;\mathrm{if}\;I_{2}\left(i,j\right)\geq 1\\ 0,\;\mathrm{if}\;I_{2}\left(i,j\right)=0 \end{array}\;\}$$

If there is pair of (P,Q) with *I*_2,*norm*_(*P*, *Q*) = 1, then there is a value *k*_1_ ∈ 1 … *N* that satisfies

$$I_{1,\mathrm{norm}}\left(P,k_{1}\right)\cdot I_{1,\mathrm{norm}}\left(k_{1},Q\right)>0.$$

Consequently,

$$I_{1,\mathrm{norm}}\left(P,k_{1}\right)\neq 0$$

and

$$I_{1,\mathrm{norm}}\left(k_{1},Q\right)\neq 0,$$

and therefore, peak p is connected to peak q via peak *k*_1_.

For the matrix *I*_2,*norm*_, an entry of value 1 at a certain position (P,Q) indicates that peaks P and Q are connected via at most one other peak, whereas the value 0 indicates no connection.

This procedure of squaring and normalizing is repeated until *I*_*h*,*norm*_ = *I*_(*h* - 1),*norm*_ and the various fitting compounds containing the respective spots can be read out.

#### Nonlinear least squared error fit of 2-dimensional Gaussian distribution curves in respective compounds

A Gaussian function of circular shape is characterized by

$$f\left(x,y\right)=I\cdot \exp\left(-0.5\frac{{\left(x-x_{0}\right)}^{2}+{\left(y-y_{0}\right)}^{2}}{\sigma^{2}}\right).$$

This leaves four parameters: the x- and y- coordinates of the peak (*x*_0_ and *y*_0_), the spot’s intensity *I* and its width/standard deviation *σ*.

A nonlinear least-squared error type of fit is used. Before the fit, the mean value of the background *μ*_*bg*_ must be calculated. Because all areas having a spot signal are enclosed by compound areas *μ*_*bg*_ can simply be calculated as the mean of the intensity values of all pixels that are not included inside a compound area. Therefore, the function that is actually fit to model the data is

$$f\left(x,y\right)=I\cdot \exp\left(-0.5\frac{{\left(x-x_{0}\right)}^{2}+{\left(y-y_{0}\right)}^{2}}{\sigma^{2}}\right)+\mu_{\mathrm{bg}},$$

with the 4 aforementioned parameters.

After determining the fitting compounds, each compound is fit individually; the spots of one compound are all fit at the same time, within the respective compound area.

The volume under the surface (VUS) of a Gaussian function curve, which is proportional to the number of photons detected by the camera and thus to the amount of protein at a certain location, can be calculated using the results from the fit via

$$\begin{array}{l} \mathrm{VUS}=\int_{-\infty }^{\infty }\int_{-\infty }^{\infty }I\cdot \exp\left(-0.5*\frac{{\left(x-x_{0}\right)}^{2}+{\left(y-y_{0}\right)}^{2}}{\sigma^{2}}\right)\mathrm{dx}\;\mathrm{dy}\\ \;=2\cdot \pi \cdot I\cdot \sigma^{2} \end{array}$$

#### Discrimination of false positives

Because spots can vary greatly in their size and intensity, it is difficult to define criteria for deciding whether a detected spot is a true or a false positive (i.e., whether a detected spot corresponds to a real spot). However, spots that are fitted with a very small width/standard deviation may represent camera noise because certain types of camera noise usually affect single pixels that subsequently form peaks without a broad base.

Thus, spots with *σ* ≤ 1 can be discriminated.

### Comparison of different spot quantification methods

When we applied the three quantification methods to simulated gel images containing two spots P and Q, each method yielded different values *signal*_*P*_ and *signal*_*Q*_, which correspond to the amount of protein signal in spot P and spot Q, respectively.

For the OD-method, *signal*_*P*_ and *signal*_*Q*_ represent the intensity of the brightest pixel of the respective spot; for the area-based method, they represent the sum of the pixel intensities in the respective spot areas. For our compound fitting method, these values represent the VUS of the curves calculated using the function parameters obtained by compound fitting. Consequently, there was no use in evaluating the different approaches on the basis of the absolute values of *signal*_*P*_ and *signal*_*Q*_. Instead, it was necessary to consider the quantity $\left(\frac{\mathrm{signa}l_{P}}{\mathrm{signa}l_{Q}}\right)$, because it shows how a method calculated the ratio of protein signal between the two spots. For the simulated spots, the true signal ratios can be calculated from the function parameters used for simulation.

This provided a measure *α* of the quality of the quantification, which was defined as the deviation of the calculated signal ratio from the true signal ratio:$\alpha =\left|\left(\left(\frac{\mathrm{signa}l_{P}}{\mathrm{signa}l_{Q}}\right)/\left(\frac{\mathrm{true}\_\mathrm{signa}l_{P}}{\mathrm{true}\_\mathrm{signa}l_{Q}}\right)\right)-1\right|\cdot 100\%$

For further information on the different models and parameters used for data simulation, refer to Additional file 1.

### Availability of supporting data

A ready-to-use version of this algorithm with graphical user interface as well as a command-line version was implemented for MATLAB (Mathworks). The code is included to the article in Additional file 2.

## Results

### The compound fitting algorithm

We developed an algorithm for the detection and quantification of protein spots on two dimensional gel electrophoresis (2-DE) images. Spot quantification is performed by fitting of 2-dimensional Gaussian function curves to the spots and calculating the volume under the surface (VUS) of the fitted functions. Before fitting, the spots are divided into fitting compounds. Only groups of neighboring spots, that show some grade of overlapping, are fitted simultaneously. The different compounds are fitted sequentially, in order to reduce the calculation time. For the determination of the minimal required compound area size, an evaluation of the fitting performance on asymmetric spots and an comparison regarding computation time between compound fitting and common fitting procedures, please refer to Additional files 3 and 4.

### Comparison of different approaches of spot quantification using synthetic data

Next, we compared compound fitting with other quantification methods. We chose to compare the following three methods for spot quantification:

#### Method 1: Optical density (OD)

The intensity of the most intense pixel of a spot, i.e. the height of the peak, is used as the measure of signal quantity. This approach is used in recent approaches [8,20] and has been reported to be a better [21] as well as a worse [22] measure of spot quantity than the area-based volume. It has been argued that OD is suitable for the resolution of overlapping spots, because it only takes into consideration the peak/center of a spot, where the contribution of overlapping parts of neighboring spots is expected to be small [23].

#### Method 2: Area-based approach

In an area-based approach, a certain area of the image that is assigned to each protein spot is used as the base for quantification. This is the most common method for spot quantification and usually the sum of the intensities of the pixels in an 'area’ , the so called 'volume’ , is used as the measure for spot signal [4,6,7,20]. Our example algorithm determined these areas according to the following scheme. First, the area of the protein signal was determined using all pixels that surpassed a certain intensity threshold. Assigning areas to spots always requires a somewhat arbitrary threshold because spots usually are continuous structures without abrupt edges that would justify borders of the area. If two spots were present in one area, it had to be decided which pixel belonged to which spot. For this decision, the minimum of the line profile between the two spots (P,Q) was calculated, and a perpendicular to the inter-peak line was drawn through the minimum; pixels on one side of this perpendicular were assigned to spot P, and pixels on the other side were assigned to spot Q. Finally, the 'volume’ was calculated by summing the pixel intensities of each area.

#### Method 3 Fitting of 2-dimensional Gaussian function curves to determine the parameters

**Figure 2:**
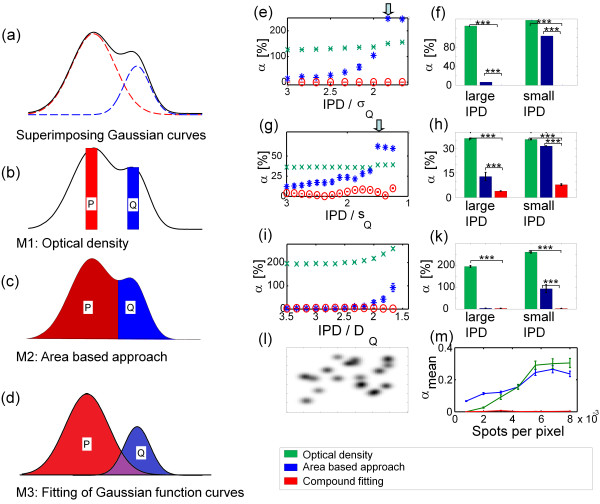
**Comparison of the different quantification approaches on simulated data sets – spot superposition. (a)** Line profile of the inter-peak line of two superimposed simulated spots of Gaussian shape. Dashed lines: superimposed curves. Solid line: resulting signal. **(b)** - **(d)** Depiction of measures for spot quantification yielded by the three methods **(b)** The OD-based approach uses the heights of the spots for quantification **(c)** The area-based approach estimates the volumes of the spots over the assigned areas. In this two-dimensional scheme, the volumes are depicted as areas P and Q. **(d)** In our method, the function parameters of the fitted curves are used to calculate the VUSs of the spots. **(e,g,i)** Performance of the different quantification methods for two representative Gaussian-shaped **(e)**, Lorentz-shaped **(g)** or diffusion model-based **(i)** spots at different extents of spot superposition. The error of quantification *α* (as defined in the methods section) increases with decreasing IPD for all quantification approaches. However, compound fitting is most robust to spot overlapping. At *IPD* ≈ 2 ⋅ *σ*_*Q*_, an increasing deterioration can be noticed (arrow, see text). 200 images per data point. **(f,h,k)** Our method yielded a significantly smaller error of quantification than the two other approaches for Gaussian-shaped **(f)** and Lorentz-shaped **(h)** spots at small (IPD = 2σ_Q_, 1.6s_Q_ or 1.7D_Q_) and large IPD (IPD = 3.5σ_Q_, 3s_Q_ or 3.5D_Q_), but only at small IPD for diffusion model-based spots **(k). (l)** Example of simulated image area containing several Gaussian spots with a range of parameters **(m)** Performance of the three quantification methods on simulated images with an increasing density of Gaussian spots. α_mean_ is a measure for quantification similar to α, adapted for the evaluation of several spots. (***: p < 0.001, unpaired two-sample t-test with Bonferroni correction). For details on data simulation, refer to Additional file 1.

The three methods are illustrated in Figure 2a-d.

### The quantification of overlapping spots

Having defined the three different quantification methods and a measure for the quality of quantification *α* (see 'Material and methods’), we compared the three methods regarding their performance in the quantification of overlapping spots. Despite the fact that the use of 2-D Gaussian functions to model protein spots is disputed [1,11],a function fit as a way to quantify protein spots has some advantages regarding spot superposition. In superimposed areas, one pixel can be influenced by several spots. While area-based methods can only assign such a pixel to one spot and thereby neglect its amount of intensity related to other spots, spot superposition can be resolved by a function fit. For the comparison we simulated gel images containing two spots with a varying grade of superposition. Taking into account that the use of 2-D Gaussian functions as a model for protein spots is disputed, we simulated not only Gaussian spots but also spots with a shape based on Lorentz curves or based on a diffusion model.The former have been discussed as a function that models the shapes of spots in 1-D electrophoresis gels [24] and the latter have been suggested as another potential function modeling 2-DE spots [11]. For all three simulated spot shapes, the results were similar. The OD miscalculated the spot ratio even at high IPD, but the result was relatively robust to spot overlap. The area-based approach yielded good results for high IPD values, but failed to resolve overlapping spots. Only compound fitting yielded satisfactory results for high and low IPD values, not only for Gaussian shaped (Figure 2e,f), but also for Lorentz-shaped (Figure 2g,h) or diffusion model-based (Figure 2i,k) spots. Importantly, the same level of precision of our method when applied to Gaussian spots could be achieved neither for Lorentz-shaped spots nor for diffusion model-based spots. With increasing spot superposition, there was a sudden large increase in *α* for the area-based approach, as shown with an arrow. At this point, the two spots were superimposed in such a way that there was no valley present on the line profile from peak P to peak Q, but the line profile was a monotonically decreasing curve and Q was the minimum. This led to further deviations in the assignment of the areas because the line used to separate the areas corresponding to each spot was drawn through the peak coordinates of Q. Remarkably, spot detection in these cases still succeeded to detect two separate spots, because peak detection was not performed on the original but rather on the restored image *A*_*res*_ (see Image preparation).The ability of a method to correctly quantify overlapping spots directly influences the method’s ability to quantify areas with a high spot density. Indeed, when we simulated images with an increasing number of Gaussian spots of various parameters (Figure 2l), the result was similar to the studies with only two spots (Figure 2m). The area-based approach yielded good results for low spot density, but the quality deteriorated for higher spot densities. The OD misestimated spot ratios, but was affected less than the area-based approach by increasing spot density. Compound fitting yielded good quantification for low and high spot density.

In summary, compound fitting proved to be a valid method for the quantification of overlapping spots, while area-based approaches faced problems especially at low inter peak distances. This effect was also observed for not-exactly Gaussian shaped spots.

### The quantification of spots with different intensities

**Figure 3:**
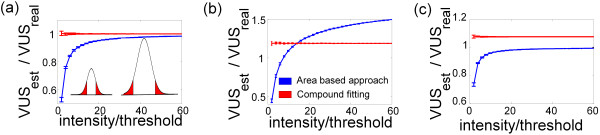
**Comparison of the different quantification approaches on simulated data sets – spots of varying height. (a)** Performance of area-based and function fit approaches for simulated single Gaussian-shaped spots of varying intensity. The function fit yielded correct VUSs for all intensities, although the area-based approach increasingly underestimated the volume at lower intensities. The intensities are indicated as multiples of the threshold that was used for defining the areas in the area-based approach. The inlay shows representative line profiles of relatively high- and low-intensity spots. The red area corresponds to the periphery of the spot, which would be omitted by an area-based approach. For the spot with the lower intensity, the red area comprises a higher percentage of the overall signal of the spot. 200 images per data point. **(b)** Performance of the area-based and function fit approaches for simulated single Lorentz-shaped spots of varying intensity. While our method consistently overestimated the VUS by approximately 20 percent, the area-based approach yielded an increasing level of underestimation at decreasing spot intensities. 200 images per data point. **(c)** Performance of the area-based and function fit approaches for simulated single diffusion model-based spots of varying intensity. While our method consistently overestimated the VUS by approximately 5 percent, the area-based approach yielded an increasing level of underestimation at decreasing spot intensities. 200 images per data point.

Furthermore, we compared the methods regarding their performance in the quantification of spots of different intensities. The use of area-based approaches is questionable because the shapes of both protein spots and Gaussian function curves do not have any abrupt edges but the intensity values decline continuously with distance from the spot’s center. Therefore, arbitrary thresholds are needed to assign a limited area to a protein spot without well-defined edges. As area-based approaches by definition evaluate spots based on limited areas, they always neglect a certain portion of the generally infinite spatial extent of a spot; i.e., they omit the periphery of a spot. This effect generally does not pose a problem in the comparison of similar spots because the measure for each spot is affected equally, and thus, the ratio of the spots remains the same. However, this ratio can be affected when comparing spots of varied intensities. In our area-based approaches, the area corresponding to a protein spot was defined by all pixels that surpass a certain fixed intensity threshold. In this manner, larger areas are assigned to spots with a higher intensity. Although it may intuitively appear correct to assign larger areas to brighter spots, this method omits a larger percentage of the signal of small spots than that of spots with a high intensity (Figure 3a, inlay) and thus might compromise the comparison of spots with different intensities.To compare the three methods in the quantification of spots with different intensities and to test this hypothesis, we simulated gel images containing spots of varying intensities and compared the volume calculated by the quantification approaches to the true spot signal. We used Gaussian-shaped (Figure 3a), Lorentz-shaped (Figure 3b) and diffusion model-based (Figure 3c) spots in the simulation. For all three types of spots, the area-based approach increasingly underestimated the spots with lower intensities, in accordance with the above explanation. The function fit overestimated the Lorentz-shaped spots and the diffusion model-based spots by approximately 20 percent or 8 percent respectively, which is readily comprehensible because Lorentz curves and diffusion model-based curves are different from Gaussian curves. However, the advantage of our method over the area-based approach was that our method overestimated all spots by a similar percentage, therefore enabling unbiased comparisons of spots with different intensities. It should be mentioned that this problem with area-based approaches is not an issue of poor assignments of the areas but is inherent to the system of evaluating curves with an infinite spatial extent based on finite areas. For example, instead of defining the areas using an intensity threshold, one could use an equally sized area for every spot (e.g., a circle around the peak with a fixed radius). This approach would solve the difficulties associated with comparing spots of different intensities but would compromise the comparison of spots with different widths.

The OD does not calculate a volume, but takes the intensity as a measure of spot signal. Therefore it could not be included into this experiment, because we compared the volume yielded by the methods to the true volume. However, as the parameter relevant for the OD is the height of the spot and this is the only parameter that is varied in this experiment, the OD perfectly depicted the ratio between spots of varying height (data not shown).

In conclusion, the area-based method biased the quantification of spots with different intensities, because it omitted a larger part of the signal for small spots than for large spots. For Gaussian as well as not-exactly Gaussian shaped spots, the compound fitting of Gaussian function curves did not show such a tendency.

### Comparison of different approaches of spot quantification using real data

We chose Aβpeptides as our primary study system for the evaluation of the different spot quantification methods. The immunoblots used differ from average 2-DE images in terms of spot number, with the immunoblots containing relatively few spots. The application of our method to a large 2-DE image containing about 1000 spots and the comparison with the noncommercial methods BEADS [4] and pinnacle [8] and the commercial software package Melanie 7 (Geneva Bioinformatics SA) can be found in Additional files 5 and 6.

### Precision of the different quantification methods

**Figure 4:**
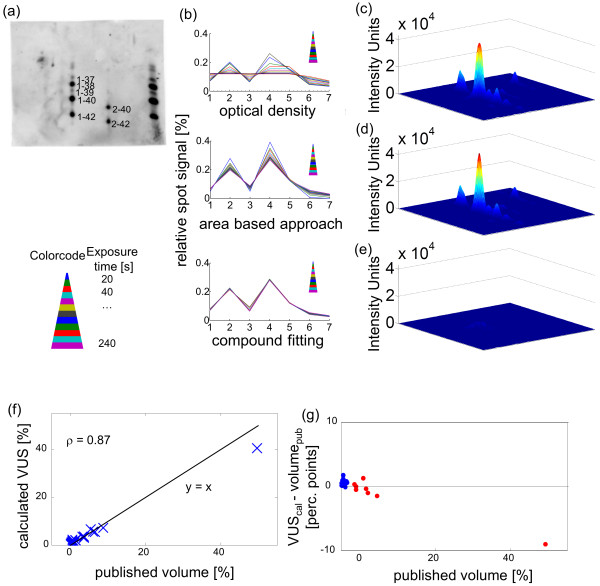
**Application of compound fitting on real data. (a)** One example blot of four blots of Aβ standard solutions. Aβ1–37/39 (30 pg, each), Aβ1–38/42 (60 pg, each), Aβ1–40 (120 pg), and Aβ2-40/42 (60 pg, each). **(b)** For one example of the four blots, the quantification of all images of the exposure time series is show. The relative spot signal (contribution of the spot signal to the overall signal of all spots combined) of the spots 1-7 was calculated in each of the 12 image of the exposure time series. One color is representative for one exposure time. It can be observed that compound fitting yielded the smallest variance of relative spot signal across the exposure time series. **(c)** 3-D surface plot of immunoblot image of Aβ peptides from human plasma. **(d)** Surface plot of the image calculated from the fitted Gaussian functions. **(e)** Residual image, difference between original and fitted image. **(f)** Correlation of the published volumes with the VUSs calculated from the fitted functions. The relative contribution of each spot to the over-all signal of the image (“total Aβ immunoreactivity“) is displayed. The published relative volumes for the 30 spots correlate well with our results, with a spearmancorrelation coefficient of ρ = 0.87. The black curve is the angle bisector. **(g)** Comparison of published volumes and calculated VUSs as a function of the spot intensity. With respect to the 8 most intense spots (red), our method yielded smaller relative VUS values than were published for 6 out of 8 spots, and higher values were obtained for 2 spots. In contrast, of the 22 lower-intensity spots (blue), our method calculated lower VUSs relative to the published data only for one spot, whereas it found the other 21 spots to be more intense than the published data.

We further evaluated our method on real data. To create a standard for the evaluation of our method, 2-D Aβ Western immunoblot was performed on Aβ standard solutions with fixed amounts of the different Aβ species. 2-DE was performed on Aβ standard solutions, the peptides were transferred to PVDF membranes and detected by a Horseradish peroxidase coupled antibody reaction (n = 4). Images of the blots (Figure 4a) were taken at different exposure times.We then tested the different quantification approaches on the images of the exposure time series. There were seven relevant spots on every blot, because the standard solution contained seven Aβ species. For each image of an exposure time series, we calculated the contribution of each spot (relative spot signal) to the sum of the overall signal of all spots (“total Aβ immunoreactivity“) in that image. This is depicted for one blot in Figure 4b. Subsequently, we determined for each spot the coefficient of variation (CV) of the relative spot signal across the exposure time series. Since the images of an exposure time series depict the same blot, a sound quantification method is expected to yield little variance of the relative spot intensities. Across all spots and blots, the mean CV of relative signal yielded by compound fitting was 0.043 (0.016 for the most intense, 0.065 for the least intense spot). For the OD and the area-based approach, the mean CV was 0.305 (0.240 for the most intense, 0.295 for the least intense spot) and 0.298 (0.208 for most intense, 0.317 for least intense spot) respectively. However, some of the images of the exposure time series were saturated. The OD is clearly not suitable for the analysis of saturated spots, because the OD of saturated peaks will always be the saturation threshold (as can be seen in Figure 4b). We therefore repeated the analysis, taking into consideration only the unsaturated images of each exposure time series. For the unsaturated images, the mean CV of the relative signal across all spots and blots was 0.007 for compound fitting, 0.044 for OD and 0.140 for the area-based approach.

In summary, for different images of the same blot, the spot intensities yielded by compound fitting have less variance compared to OD and area-based approach. When saturated images were included, the performance of OD and the area-based approach were similar. However, when only unsaturated images were analyzed, OD was superior to the area-based approach.

### Accuracy of the different quantification methods

Having evaluated the precision of our method, we also wanted to measure the accuracy of compound fitting, i.e. the grade to which the results display the actual protein amounts. For each blot, we used the most intense image that did not show any saturation. We compared only the Aβ_1–37/38/39/40/42_ spots in our standard immunoblots, because the primary antibody (mAb 1E8) used for staining binds to the N-terminus and the differential binding of the antibody compromises the comparison of Aβ_1-x_ and Aβ_2-x_species. In all four blots, we compared the relative spot signal of an Aβ species yielded by the three quantification methods to the percentage of the amount of that species in the standard solutions (relative peptide amount). For compound fitting, the mean deviation of the relative signal to the relative peptide amount was 3.81%, for OD and the area-based approach the mean deviation was 4.37% and 4.71%, respectively.

This means that compound fitting has a 13% or 19% higher accuracy than OD or the area-based approach, respectively. However, one has to be cautious when comparing the protein amount with the spot signal. Differential binding of the antibody, solubility of the different species, possible oxidation reaction and many more variables can influence the relation between protein load of the gel and detected signal.

### Comparison of compound fitting with published, area based, results

Next, we applied our algorithm to immunoblot images of Aβ peptides in human plasma in healthy controls using datasets which we published previously [15]. When our algorithm was applied to the original images, we found that it produced a suitable fit for the images (Figure 4c-e).In our previous publication, 30 peptide spots were identified, and the relative contribution of each spot to the sum of the overall signal of all counted spots (“total Aβ immunoreactivity“) was calculated using an area-based approach (Quantity One software v4.1, BioRad). The relative spot signals calculated by our method closely matched the published values (spearman’s rho ρ =0.87, Figure 4f).Interestingly, the differences between the published area-based results and the results of the fit of the corresponding images showed a systematic trend. For spots with a low intensity, our method yielded higher relative values than the area-based approach, whereas for the spots with the highest intensities, the area-based approach yielded higher values (Figure 4g). This finding is in accord with our simulations on spots with different intensities (Figure 3). In the presented data, the peak intensity values varied by the factor 100 from lowest to highest. Because low-intensity spots are underestimated by the area-based approach but not by the function fit method, the area-based approach calculates a lower contribution to the total Aβ immunoreactivity for such spots. Consequently, because the sum of all spots must be equal to 100%, the area-based approach overestimates spots with a high intensity.

In summary, area-based approaches might underestimate small spots in immunoblot images of Aβ peptides in human plasma.

## Discussion

In this present work, we validated the compound fitting of two dimensional Gaussian functions as a method for the quantification of two dimensional gel electrophoresis (2-DE) data, thereby enabling robust comparisons between different protein spots within a brief computation time. The fitting method was found to be valid for quantifying simulated data and was further evaluated using immunoblots of Aβ standard solutions and a published set of 2-DE images. The comparison of the compound fitting approach to area-based quantification methods revealed two advantages of the function fit method: 1. an improved ability to resolve superposed spots and 2. The unbiased comparison of spots with greatly differing heights/widths. The optical density (OD), i.e. the intensity of the most intense pixel of a spot, as a measure for spot signal does not take into account the width of a spot and is therefore not suitable to depict the signal ratio between different spots. However, it perfectly depicts the ratio between spots of similar shape but different height and is relatively robust to spot overlapping. It might therefore be useful for differential expression analysis across several gels, especially if the spots are strongly distorted. When we applied all three methods to exposure time series and standard gels of Aβ peptides, compound fitting showed the best precision as well as accuracy. Although for our data, our method produced suitable fits with little residues (Figure 4c-e), it has also been argued that protein spots are often not modeled exactly by Gaussian spots [11]. However, even for not-exactly-Gaussian spots, if the underlying function is not known, compound fitting may be the method of choice due to the aforementioned advantages, as demonstrated by the application of our algorithm to Lorentz-shaped or diffusion model-based spots. Although, as expected, our algorithm miscalculated those spots, it nevertheless outperformed the area-based approaches in terms of the resolution of superimposed spots (Figure 2e-m) and the comparison of spots of varying height (Figure 3). Due to these advantages, fitting of 2-dimensional Gaussian function curves has a higher precision (Figure 4b) and accuracy than area-based approaches when evaluated on immunoblots of standard peptide solutions.

## Conclusion

Low SNRs and heterogeneous image conditions across different gels are common problems in 2-DE data analysis [7]. We found that it was most practical to robustly detect peaks and then allow the user to inspect the results and add or discard peaks based on experience. Spots that are clearly separated in one experiment may not be detected as separate spots in another gel. In this case, the experienced user might add or correct peak locations. However, because all spot detection methods have advantages and drawbacks when addressing a wide variety of electrophoresis images [1], compound fitting as a quantification tool could be combined with other spot detection algorithms or the manual identification of spots. This combination and the influence of different spot detection methods on the quantification process are described in Additional file 7.

With respect to Aβ peptides, high-quality quantification is necessary to use these peptides as biomarkers [17]. In addition, the exact quantification of 2-DE spots will continue to be crucial for the identification of differences in the amounts of Aβ species in the plasma of patients with Alzheimer’s disease and for the development of additional molecular markers.

## Abbreviations

2-DE: Two dimensional gel electrophoresis; CV: Coefficient of variation; IPD: Inter peak distance; OD: Optical density; SNR: Signal to noise ratio; VUS: Volume under surface.

## Competing interests

The authors declare that they have no competing interests.

## Authors’ contribution

JMB and TWG conceived the study, evaluated the algorithm and wrote the manuscript. JMB, AS and SG implemented the algorithm. TO performed the experiments. JW, JK and JMM conceived the experiments. All authors read and approved the final manuscript.

## Supplementary Material

Additional file 1 Supplementary methods: Data Simulation in detail. Click here for file

Additional file 2 Matlab code: Compound fitting. Click here for file

Additional file 3 Evaluation of compound fitting on synthetic data. Click here for file

Additional file 4: Figure S1 Performance of compound fitting on simulated data sets. (a) Influence of compound area size and SNR on the quality of fit. For each curve, the deviation of the fit result from the true VUS is displayed for varying compound area size d. For comparison, the curves for the different SNRs are plotted above each other. The inlays show examples of simulated spots with respective SNR.200 images per data point. (b) Evaluation of the quality of fit on asymmetric Gaussian-shaped spots. The deviation of the fit result from the true spot signal is displayed as a function of the asymmetry of the spot. The inlays show simulated spots with *σ*_*x*_/ *σ*_*y*_ = 1, 2 and 3, from left to right. (c) Comparison of compound fitting and usual fitting. The computation time in seconds for compound fitting (red) and usual fitting (blue) is displayed in dependence of the number of simulated spots in the image. 200 images per data point. For details on data simulation, refer to additional file 1. Click here for file

Additional file 5 **Analysis of a large 2-DE image and comparison with other methods **[25]**,**[26]**.** Click here for file

Additional file 6: Figure S2 Immunoblot of Aβ peptides in human plasma from healthy controls. Click here for file

Additional file 7 The combination of compound fitting with different spot detection methods. Click here for file
