# Supplementary material for: Spot quantification in two dimensional gel electrophoresis image analysis: comparison of different approaches and presentation of a novel compound fitting algorithm
Source: BMC Bioinformatics. 2014 Jun 11;15:181. doi: 10.1186/1471-2105-15-181 (PMC4085234; doi:10.1186/1471-2105-15-181)
Supplement: Additional file 1 — Supplementary methods: Data Simulation in detail. [file 1471-2105-15-181-S1.doc]

**Supplementary methods: Data Simulation in detail**

*Equations for different spot models*

For the simulation of spots we used three different models:

2-dimensional Gaussian function curves, given as

$$f(x,y)=I\cdot\exp\left(-0.5\frac{\left(x-x_{0}\right)^{2}+\left(y-y_{0}\right)^{2}}{\sigma^{2}}\right),$$

two dimensional Lorentz function curves, given as

$$f\left(x,y\right)=I\cdot\frac{s}{\left(\left(x-x_{0}\right)^{2}+\left(y-y_{0}\right)^{2}+s^{2}\right)^{1.5}},$$

with a height-defining parameter *I*, a width-defining parameter *s* and the peak coordinates $x_{0}$ and $y_{0}$,

and spots based on a diffusion model[11], given as

$$f(x,y)=\frac{C_{0}}{2}\left[erf\left(\frac{a'+r'}{2}\right)+erf\left(\frac{a'-r'}{2}\right)\right]$$

$$+\frac{C_{0}}{r'\cdot\sqrt{\pi}}\left[\exp\left(-\left(\frac{a'+r'}{2}\right)^{2}\right)+\exp\left(-\left(\frac{a'-r'}{2}\right)^{2}\right)\right],$$

with$r'=\sqrt{\frac{\left(x-x_{0}\right)^{2}}{D_{x}}+\frac{\left(y-y_{0}\right)^{2}}{D_{y}}}$, a height-defining parameter*I*, the diffusion derived width defining parameters $D_{x}$ and $D_{y}$, the area of the disc from which the diffusion process starts $a'$and the peak coordinates $x_{0}$ and $y_{0}$.

For the Gaussian functions, the VUS can be calculated as

$$VUS=\int_{-\infty}^{\infty} \int_{-\infty}^{\infty} I\cdot\exp\left(-0.5*\frac{\left(x-x_{0}\right)^{2}+\left(y-y_{0}\right)^{2}}{\sigma^{2}}\right)dx dy=2\cdot\pi\cdot I\cdot\sigma^{2}$$

For Lorentz functions, the VUS can be calculated as

$$VUS=\int_{-\infty}^{\infty} \int_{-\infty}^{\infty} I\cdot\frac{s}{\left(\left(x-x_{0}\right)^{2}+\left(y-y_{0}\right)^{2}+s^{2}\right)^{1.5}}dx dy=\pi\cdot I$$

For the diffusion-based model, the VUS was calculated by numeric integration.

*Parameters for simulated images*

The background of the images was modeled as having a constant intensity $\mu_{bg}$and Gaussian distributed noise with mean of 0 and a standard deviation $\sigma_{ns}$.

To test the influence of the compound area size on the quality of thefit (Additional file 1: Figure S1a), we simulated images containing one single Gaussian shaped spot ($I=20000$,$\sigma=5$).

The SNR of a spot was defined as:$SNR=\frac{I}{\sigma_{ns}}$,where*I* is the height of the peak above the background[5].

To compare compound fitting to usual fitting (Additional file 1: Figure S1b), we simulated images with varying numbers of Gaussian shaped spots of a wide range of parameters.

For the comparison of the different quantification approaches (Figure 2e-k), we simulated three data sets. For one set of gel images we simulated pairs of superimposed Gaussian function curves, whereas we used Lorentz-shaped or diffusionmodel-based spots for the other sets. For the bar graphs (Figure 2f,h,k), superimposed spots with varying IPD were simulated for a wide range of function parameters.

The exemplar spots evaluated in Figure 3e,g,ihad the following parameters:

$I_{P}=20000, I_{Q}=12000, \sigma_{P}=4, \sigma_{Q}=6$for the Gaussian and

$I_{P}=20000, I_{Q}=16000, s_{P}=10, s_{Q}=8$for the Lorentz shaped spots.

$C_{P}=100000, C_{Q}=10000, D_{P}=4, D_{Q}=6,a'{}_{P}=1,a'_{Q}=3$for the diffusion model-based spots.

The parameters of the spots in images simulated for Figure 3l,m were as follows:

$I=5000 to 10000, \sigma= 4 to 6$. The locations of the spots were randomly distributed.

For the quantification analysis of spots with different intensities (Figure 3n,o,p) we simulated Gaussian/Lorentz shaped or diffusion model-based spots of varying intensity, with$\sigma=5, s=5, D=6,a'=3$.
