# Supplementary material for: Spot quantification in two dimensional gel electrophoresis image analysis: comparison of different approaches and presentation of a novel compound fitting algorithm
Source: BMC Bioinformatics. 2014 Jun 11;15:181. doi: 10.1186/1471-2105-15-181 (PMC4085234; doi:10.1186/1471-2105-15-181)
Supplement: Additional file 3 — Evaluation of compound fitting on synthetic data. [file 1471-2105-15-181-S3.doc]

**Evaluation of compound fitting on synthetic data**

**a) Determination of compound area size**

The size of the compound areas, and therefore the compounds, affects the quality of the fit but also the computation time. A compound area must be sufficiently large to guarantee an optimal fitting result but sufficiently small to allow the fit to be performed within a reasonable amount of time.

To determine the minimum compound size required for optimal fitting, we simulated images with single Gaussian spots at various SNR. We analyzed the images by compound fitting Gaussian function curves using various compound sizes *d* (figure S1a). The deviation of the fit result from the true VUS was used as the measure for the quality of the fit. We found that spots with a low SNR required a larger compound area size to be fit accurately. However, provided that *d* was at least $2\cdot\sigma$, the quality of the fit remained unaffected for all SNRs.

For real data, $\sigma$of the spots is not known. If *d* is set too low, it compromises the quality of the fit, whilst a high value for *d* might compromise the computation time but not the quality of the fit. For the application on real data, the value for *d* is set as follows:

A line profile through the peak of the most intense spot ofthe image can be fitted with a 1-D Gaussian function. If *d* is set as$d=4\cdot\sigma$, with $\sigma$ being the standard deviation of the fitted 1-D Gaussian function, *d* is sufficiently large for optimal fitting.

In conclusion, the quality of the fit is robust with respect to variations in noise but requires sufficient compound area size.

**b) Evaluation of fitting performance on asymmetric spots**

As not all protein spots are completely symmetric, we evaluated the performance of compound fitting on asymmetric spots. We simulated Gaussian spots with varying grades of asymmetry ($\sigma_{x}/ \sigma_{y}$) and compared the VUS yielded by the fit with the true spot signal calculated from the simulation parameters (figure S1b). Spots with an aspect ratio > 2:1 should not be evaluated by fitting of symmetric Gaussian function curves, in order to keep the error of estimation below 5%. However, as can be seen in the inlays in figure S1b, such spots are unusual and it has to be carefully considered whether they represent a single spot or rather a complex of multiple protein spots.

**c) Comparison of compound fitting and usual fitting**

One of the advantages of compound fitting over usual fitting, i.e. fitting all spots at once, is its computational efficiency. To study this effect, we simulated gel images containing a variable number of Gaussian spots with diverse function parameters. To these images, we applied our compound fitting algorithm and a usual fitting algorithm that fits all spots and pixels at the same time. For both algorithms, we measured the computation time and, as a measure for the quality of the fit,the deviation of the fit result from the true VUS. In both cases, over 99 percent of the computation time was required for the actual fitting routine and all other parts of the algorithm (preparatory steps, determination of the fitting compounds,…) required less than one percent of the overall computation time. For usual fitting, the computation time increased heavily with the number of peaks, while for compound fitting it remained relatively constant (figure S1c). In the immunoblots in[15], which we also used for further evaluation below, 30 Aβspecies were identified. The usual fitting algorithm needed 135 seconds to analyze one image with 30 spots, but the compound fitting method needed 7 seconds. No significant difference in the quality of fit could be observed between the two methods (data not shown).

So in conclusion, compound fitting is much more computational efficient for higher spot numbers and density than usual fitting.
