# Supplementary figures and images for: Spot quantification in two dimensional gel electrophoresis image analysis: comparison of different approaches and presentation of a novel compound fitting algorithm
Source: BMC Bioinformatics. 2014 Jun 11;15:181. doi: 10.1186/1471-2105-15-181 (PMC4085234; doi:10.1186/1471-2105-15-181)

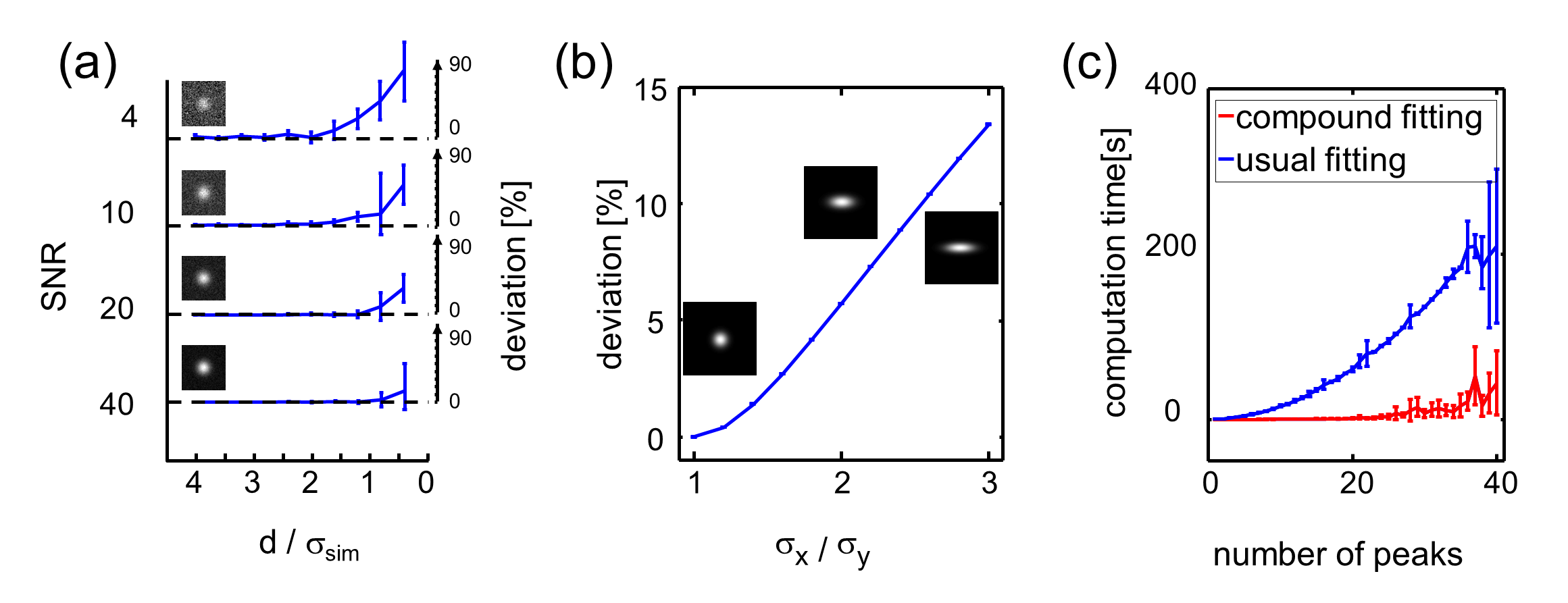

Supplement: Additional file 4: Figure S1 — Performance of compound fitting on simulated data sets. (a) Influence of compound area size and SNR on the quality of fit. For each curve, the deviation of the fit result from the true VUS is displayed for varying compound area size d. For comparison, the curves for the different SNRs are plotted above each other. The inlays show examples of simulated spots with respective SNR.200 images per data point. (b) Evaluation of the quality of fit on asymmetric Gaussian-shaped spots. The deviation of the fit result from the true spot signal is displayed as a function of the asymmetry of the spot. The inlays show simulated spots with σ x / σ y = 1, 2 and 3, from left to right. (c) Comparison of compound fitting and usual fitting. The computation time in seconds for compound fitting (red) and usual fitting (blue) is displayed in dependence of the number of simulated spots in the image. 200 images per data point. For details on data simulation, refer to additional file 1. [file 1471-2105-15-181-S4.tiff]

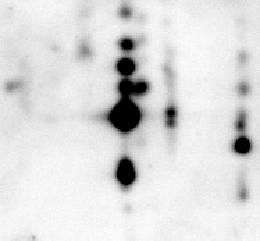

Supplement: Additional file 6: Figure S2 — Immunoblot of Aβ peptides in human plasma from healthy controls. [file 1471-2105-15-181-S6.png]
