# Supplementary material for: Spot quantification in two dimensional gel electrophoresis image analysis: comparison of different approaches and presentation of a novel compound fitting algorithm
Source: BMC Bioinformatics. 2014 Jun 11;15:181. doi: 10.1186/1471-2105-15-181 (PMC4085234; doi:10.1186/1471-2105-15-181)
Supplement: Additional file 5 — Analysis of a large 2-DE image and comparison with other methods [25],[26]. [file 1471-2105-15-181-S5.doc]

**Analysis of a large 2-DE image and comparison with other methods**

We wanted to test the performance of compound fitting on a typical 2-DE image containing many hundreds of spots. We chose to analyze an image that had been used by others for the evaluation of 2-DE image analysis software [25,26]. The image is available online at http://www.umbc.edu/proteome/image_analysis.html (gelb). As it is not possible to know the ground truth, i.e. the true spot volumes, in an image like that, we decided to evaluate the performance of our compound fitting algorithm by comparing it to several established methods. The methods chosen for comparison are the noncommercial applications pinnacle [8] and BEADS [4], and Melanie 7 (Geneva Bioinformatics SA), one of the most popular commercial software packages. BEADS is a surface-oriented method that detects spots by inverting the image and then following the flow of virtual beads along intensity gradients to the sinks of the inverted image. It quantitates the spots with the measure ‘volume’ calculated with an area-based approach. Virtual beads start rolling from every pixel and the area is defined as the region of pixels whose beads end up in peak of the spot. Pinnacle uses local maxima for spot detection and the optical density (OD), for quantification. Both are non-commercial and available from the authors upon request. It is not stated in the user manual how the spot detection algorithm of Melanie works. The primary measure for spot quantification is the volume yielded from an area-based approach, but it is also possible to quantitate spots by OD. All spot detection methods required user intervention, so we tried to optimize the detection parameters. BEADS was executed using standard parameters, as variation of the parameters did not considerably affect the result. Pinnacle yielded many false positive spots (8200 spots on the whole image) when executed with standard parameters, so the parameters were adjusted. Melanie 7 does not offer any standard detection parameters, so the parameters were also adjusted. For the comparison of spot quantification, we considered the spots that were detected by both our method and the respective other approach. Spots were counted as commonly detected, if their peak coordinates yielded by our method and the respective other method deviated no more than four pixels. The result of the experiment is shown in table 1. It is obvious that the quantification by compound fitting correlated better with the area-based approaches than with the OD approaches. We then analyzed only the spots that were detected by every method, and calculated the pairwise correlation of quantification for all methods (table 2). The highest correlation was found between the two OD-based approaches (pinnacle and the OD measure of Melanie). The two area-based approaches (BEADS and the volume measure of Melanie) also correlated well. Compound fitting showed a medium correlation with all four other quantification methods, while the two area-based approaches showed the least correlation with the two OD-based measures.

**Table 1** **Comparison of spot detection and quantification between compound fitting and other methods.**

|  | Spots found | Spots matched | Pearson‘s r |
| --- | --- | --- | --- |
| BEADS | 319 | 274 | 0.87 |
| pinnacle | 1433 | 659 | 0.73 |
| Melanie - OD | 1081 | 744 | 0.74 |
| Melanie - volume | 1081 | 744 | 0.84 |
| compoundfitting | 895 | - | - |

Spots found: total number of spots found in the image; spots matched: number of spots commonly detected by our method and the respective method; Pearson’s r: correlation between the quantification of the commonly detected spots (normalized to the overall spot signal in the image) between our method and the respective method.

**Table 2** **The quantification of commonly detected spots yielded by the different methods.**

|  | BEADS | pinnacle | Melanie - OD | Melanie - volume | compound fitting |
| --- | --- | --- | --- | --- | --- |
| BEADS | 1 | 0.79 | 0.77 | 0.95 | 0.88 |
| pinnacle | - | 1 | 0.99 | 0.75 | 0.82 |
| Melanie - OD | - | - | 1 | 0.76 | 0.81 |
| Melanie - volume | - | - | - | 1 | 0.86 |
| compound fitting | - | - | - | - | 1 |

Pairwise Pearson’s correlation coefficient for the quantification measures yielded by the different methods**.** Only the spots that were detected by every method were taken into account.
