# Supplementary material for: Spot quantification in two dimensional gel electrophoresis image analysis: comparison of different approaches and presentation of a novel compound fitting algorithm
Source: BMC Bioinformatics. 2014 Jun 11;15:181. doi: 10.1186/1471-2105-15-181 (PMC4085234; doi:10.1186/1471-2105-15-181)
Supplement: Additional file 7 — The combination of compound fitting with different spot detection methods. [file 1471-2105-15-181-S7.doc]

**The combination of compound fitting with different spot detection methods**

We wanted to evaluate the performance of the quantification by compound fitting when combined with various spot detection methods. Different spot detection methods are not expected to have a big impact on spot quantification for two reasons: 1. The function fitting is performed on the original image, preprocessing is only performed for the peak detection. 2. The peak coordinates of the fitted Gaussian curves are allowed the deviate to a certain degree from the peak coordinates suggested by the spot detection, thus eliminating impact of small changes in the detected peak coordinates on the quantification. However, if one of two superimposing spots is not detected, this will affect the quantification of the detected spot.

We used three further spot detection methods: BEADS [4] is a surface-oriented method that detects spots by inverting the image and then following the flow of virtual beads along intensity gradients to the sinks of the inverted image. RegStatGel [3] is a segmentation-oriented method that uses the watershed transformation. Both are non-commercial and available from the authors upon request. The third method we compared was Melanie 7 (Geneva Bioinformatics SA), one of the most popular commercial image analysis suites. It is not stated in the user manual how the spot detection algorithm works. We applied the four spot detection methods to an immunoblot image of Aβ peptides in human plasma in healthy controls (figure S2). 30 Aβspots have been identified in this image by an expert [15]. As all spot detection methods require user intervention, we tried to optimize the detection parameters to have as many as possible of the 30 spots detected. For our method, we set the threshold parameter t to 3 (instead of 10, as was used for the other analyses), because the image was recorded with low exposure time and was quite faint. Melanie 7 requires three parameters for spot detection and does not offer any predefined standard settings, so we optimized those three parameters. BEADS and RegStatGel offer predefined detection parameters. We used the predefined settings, as variation of the parameters showed little impact on the resulting peak detection. The respective spot peak coordinates were then used for compound fitting of the image. In the case of RegStatGel, which performs segmentation of the image, but does not calculate peak coordinates, the pixel with the highest intensity in every image segment was chosen for the peak coordinates. We then took the peak coordinates yielded by the different detection methods and quantified the respective spots with compound fitting. The quantification of the Aβ peptide spots was highly correlated for the different spot detection methods, although the number of detected spots varied considerably (table 3). This means that the quantification by compound fitting is relatively robust to different spot detection methods.

**Table 3** **Correlation of the quantification of spots found by different detection algorithms but quantified with compound fitting.**

|  | Aβ peptide spots found | Total spots found | Pearson’s r |
| --- | --- | --- | --- |
| BEADS | 12 | 13 | 0.90 |
| RegStatGel | 8 | 8 | 0.98 |
| Melanie | 25 | 41 | 0.91 |
| Our detection routine | 27 | 43 | - |

Aβ peptide spots found: number of the 30 Aβ spots found by the respective method; Total spots found: total number of spots detected in the image; Pearson’s r: correlation between the quantification measures yielded by the respective spot detection routine combined with quantification by compound fitting and our method (detection + quantification)
